# Clinical Animal Behaviour: Paradigms, Problems and Practice

**DOI:** 10.3390/ani12223103

**Published:** 2022-11-10

**Authors:** Daniel S. Mills

**Affiliations:** Animal Behaviour, Cognition and Welfare Group, Department of Life Sciences, University of Lincoln, Lincoln LN6 7DL, UK; dmills@lincoln.ac.uk

**Keywords:** applied behavioral analysis, behaviorism, behavioural medicine, companion animals, evidence based medicine, scientific method, scientific philosophy, veterinary behavioural medicine

## Abstract

**Simple Summary:**

Effective and ethical management of problem animal behaviour requires the translation of scientific research into practice. However, to do this we must appreciate different scientific perspectives and their limitations. There are serious limitations to the application of population level effects to the care of the individual. Factors such as *p*-values relating to difference, or lack there of, appear to be frequently misunderstood and may be of less value than widely appreciated. Clinical significance can be very different to statistical significance. There is also a growing concern over the way in which an approach supposedly based on evidence medicine is being applied to treatment recommendations. This is in danger of creating unhelpful biases that can undermine the delivery of personalized care which is at the heart of clinical animal behaviour practice. In order to address this, increased open access and data-sharing is to be encouraged. Evidence from scientific studies needs to be combined with critical reflection of its relevance on a case by case basis by clinicians. Accordingly it is imperative that they have a high level of scientific literacy.

**Abstract:**

Both the public and clinicians are interested in the application of scientific knowledge concerning problem animal behaviour and its treatment. However, in order to do this effectively it is essential that individuals have not only scientific literacy but also an appreciation of philosophical concepts underpinning a particular approach and their practical implications on the knowledge generated as a result. This paper highlights several common misunderstandings and biases associated with different scientific perspectives relevant to clinical animal behaviour and their consequences for how we determine what may be a useful treatment for a given patient. In addition to more reflective evaluation of results, there is a need for researchers to report more information of value to clinicians; such as relevant treatment outcomes, effect sizes, population characteristics. Clinicians must also appreciate the limitations of population level study results to a given case. These challenges can however be overcome with the careful critical reflection using the scientific principles and caveats described.

## 1. Introduction

Clinical animal behaviour is the management of problem animal behaviour, and emerged as a specific scientific discipline about 50 years ago [1]. As an academic discipline with a strong practical element, it is of interest to many members of the public, researchers and clinicians; many of whom are prepared to offer an opinion on best practice. It involves an understanding of the interaction that occurs between people, their animals and their environment at both the nomothetic level (i.e., in terms of the general laws involved in behaviour, perception, etc.) as well as the idiographic level (i.e., in terms of the specific factors affecting particular individual). The scientific basis to clinical animal behaviour is multidisciplinary and different perspectives have tended to dominate at particular times as it has come to the attention of different disciplines. This has been accompanied by changing attitudes within society towards animals, their wellbeing and ethical management [2,3,4]. Accordingly, it is not surprising that there are frequent debates at the scientific, clinical and public level about what are appropriate and/or effective interventions, e.g., see the recent debate on the use of handheld electronic training collars for dogs [5,6,7].

Greater engagement with scientific research literature by clinicians has led to many benefits from a more evidence-based approach over the last 30 years [8]. Likewise, the greater democratization of science can also be a positive force [9]. However, competence in the understanding of science and the hijacking of public opinion by special interest groups are valid concerns [10]. It should also be noted that the information required by clinicians to make better judgments (such as the size of an effect) is not necessarily the same as that which is routinely published by scientists who may be more focused on establishing the statistical significance of differences between populations. A focus on statistically significant population level effects may also hide important individual differences of clinical significance [11], which might be apparent from qualitative methodologies with a greater sensitivity to idiographic information, e.g., see studies by Barcelos et al., 2015 [12] and Lopes Fagundes et al., 2018 [13].

The nature of clinical animal behaviour and especially the complex nature of many interventions, mean that important philosophical underpinnings [14] and assumptions may not be appreciated by the public, clinicians or even scientists working in the field, with poor quality conclusions drawn as a result. This can have real world impact on the standard of patient care and scientific progress in this field. Therefore, the primary aim of this article is a pragmatic one: to provide a point of reference for encouraging a more effective scientific evaluation and discussion of interventions within the field of clinical animal behaviour for those interested in the topic. However, of particular note in this regard is an appreciation of the different schools of thought concerning the nature of problem behaviour, the types of intervention favored as a result and the standards used to assess efficacy. A secondary aim is therefore more academic: to highlight the importance of appreciating the philosophical underpinnings to our perspectives on this topic, and potential limitations therein. It is hoped that this will contribute to better quality research, presented in a way that is of most use to clinicians, so that the undoubted value of a scientific approach can be more fully realized to the benefit of patients.

## 2. The Scientific Method, Objectivity and Intrinsic Biases

An essential feature of the scientific revolution which developed out of the Age of Enlightenment, is the questioning of our assumptions and beliefs within the context of current knowledge and rational argument [15]. *Scientific disagreement is common, but based on rational argument and should never be personal*. It is not about winning the argument, but rather getting to the truth. This is one of the reasons why scientists place more weight on peer-reviewed publications than what is on the wider internet, for evidence. Reliable scientific enquiry requires an appreciation of the assumptions underpinning the nature of what is being studied (ontology), and distinction between opinion and justified belief as sources of knowledge (epistemology). This demands that we give consideration to what might be accepted as factual from a scientific perspective and the methods used to generate scientific knowledge. A key feature of the scientific method is its reproducibility, and it can be argued that insufficient value has been placed on this by some scientific publishers [16,17], although the development of initiatives such as the ARRIVE (Animal Research: Reporting In Vivo Experiments, arriveguidelines.org) are a welcome development. Unless a result is reproduced the data might be flawed due to differences in practical execution. This is particularly important to consider in relation to scientific research into inferred constructs, such as psychological states or cognitive abilities [18]. From an historical perspective scientific advances and associated research initially related to our understanding of the physical world [15] but this reflects, at least in part, a cultural bias related to the areas where it was perhaps easiest/most convenient to apply the scientific method (an epistemological bias). It seems that a failure to appreciate the hegemony arising from this has led some to conclude that physical phenomena form the only basis to true knowledge [19] (a philosophical position referred to as physical reductionism) and that the scientific method can only be applied to such phenomena. Both the former ontological claim and the latter epistemological one are limiting and unsound.

In the case of problem behaviour, there are important ontological considerations relating to the nature of problem behaviour and epistemological ones relating to how we assess the underling phenomenon. The first ontological consideration relates to the concept of “a problem”; integral to this is how the actions of an animal *are being perceived and/or interpreted* by the carer who reports the problem. For example, in relation to attention seeking behaviour, what may be seen as an annoying habit or problem by one owner may be an endearing habit to another [20]. In other words, the problem is not just the observable behaviour per se, but how it is represented by the owner. Thus, if we limit ourselves to considering just the physical basis to the problem, we are only considering part of the issue. There is clearly a need to address the problem through means that extend beyond simply modifying the behaviour of the animal, e.g., by preparing the client psychologically and physically to undertake necessary behaviour change [21]. This means interventions are typically complex and multimodal, which has important implications when we consider the application of standards used in evidence-based medicine to the assessment of clinical behaviour treatment efficacy (discussed below). Secondly, even if we focus just on the behaviour of concern there are important non-physical phenomena (cognitions and/or emotions) that regulate its expression [22]. Important knowledge can be gained by representing these phenomena as intervening psychological variables (e.g., a state of fear or frustration) which have a causal relationship with the behaviour [23]. We cannot directly measure the mental states of others, but, as discussed below, it may not only be possible to logically infer mental status indirectly from other measures, but also to be “scientific” in the way we do this. To make such inferences in a scientifically justifiable way we need to be able to apply the scientific method. The scientific method involves observation, inductive reasoning to formulate hypotheses which can be tested using deductive reasoning and skepticism to reduce uncertainty [24]. The scientific method is an iterative process, built on a desire to reduce uncertainty; it does not seek to establish absolute truths—a point that is often misunderstood. Nonetheless, from an epistemological perspective it is a better source of knowledge than many others which are less objective.

The nature of the objectivity of the scientific method is widely misrepresented [25], with misleading claims that science is entirely objective; this can result in a neglect of the need for constant reflection to reduce bias. It is wrong to think of the method as completely objective or that anything that does not have a physical basis is unscientific. For example, to illustrate the former point, when we choose to study something using the scientific method, we have made a subjective decision in both what we have chosen to study and how to study it. We have taken a particular epistemological perspective and are thus not being entirely objective. Having embarked on this process, we will inevitably examine it with a certain bias related to our perceptual abilities and subjective ontological assumptions about the nature of the phenomenon being studied. Our senses and thus what we perceive about the world, are limited and focused on providing us with the information that is optimal for our survival and not unbiased physical reality [26]; i.e., we do not have an objective view of the whole world. For example, in the case of vision, it is well established that humans perceive only a small part of the electromagnetic spectrum (between about 380 and 750 nm), whereas bees and many birds perceive a different range (extending into ultraviolet), which is utilized by many flowers to provide important information that aids pollination. Only when researchers step away from studying the behaviour of other animals within the context of human sensory and cognitive abilities, do certain mysteries become potentially explainable [27].

There is also often a subjective bias towards focusing on the physical aspects of phenomena, although this (despite the claims of some scientists) is not mandated by the scientific methods as defined above. For example, the scientific study of the psychological capacities of non-human animals, has a long history of rejection of ideas by the scientific establishment [28]. In relation to problem behaviour there have historically been two main scientific perspectives [29]—a medical approach and a behavioral one (discussed below), with both of these grounded in a bias towards an emphasis on the physical basis to phenomena (physical reductionism). More recently, a third more eclectic “psychobiological” perspective has emerged [29], which seeks to embrace the value gained from inferring internal psychological states to explain the occurrence of behaviour. All three approaches may be considered scientific in that they are built on an appreciation of the value of the scientific method for establishing knowledge, but their value is at least partly defined by their utility in practice to predict and explain problem behaviour and promote humane effective management. In the next section, I briefly describe these perspectives and their associated ontological associations before focusing more on the psychobiological approach and its application in practice.

## 3. Three Scientific Perspectives on the Nature of Clinical Animal Behaviour and Some of Their Implications

The two (non-exclusive) approaches that have historically dominated our thinking and practice of clinical animal behaviour are described below and will be readily recognized by those working in the field [29]:

1. the medical model tends to view problem behaviour as a pathological state, akin to a physical disease and is grounded in a physical positivist reductionism, with the source of the problem being, for example, in a chemical brain imbalance.
2. the behavioral model emphasizes the importance of environmental contingencies in shaping behaviour. This is grounded in the behaviorism of experimental psychology, but also has a physical positivist focus that is outside the animal—on the observable events in the environment.

In the last 25 years an effort has been made to develop a third eclectic and systematic approach [29,30], this may be less familiar to the reader and so is described in a bit more detail below:

3. the “psychobiological” perspective draws on developments in affective neuroscience, behavioural biology and evolutionary biology. This also has a different ontological perspective, by emphasizing the importance of referencing a range of psychological constructs, such as motivational and emotional state, as useful variables for predicting individual behavioural responses. In this context, motivation refers to the psychobiological processes controlling actions for which there is a single explicit goal that is being pursued at a given time. The associated biological processes include the neuro-endocrinological states and responses underpinning the action; while the psychological processes include any representation of the importance and goal of the behaviour as well as the choices made. Identification of reinforcement effects (which are prominent in the behavioral model) helps in the formulation of hypotheses about the goal of the behaviour. By contrast the term emotion is used to describe a multicomponent psychobiological response arising from the perception and appraisal of the significance and importance of elements of the environment at a given time to a particular individual, in line with the component process theory described by Scherer [31]. The response is organizational and associated with a probabilistic change in a suite of behaviours associated with a particular biological function. Emotional behaviours have the characteristics of persistence, valence, scalability and generalization (emotion primitives *sensu* Anderson & Adolphs [32]) and arise due to personally significant events in the environment, and this may relate to the presence, absence or predicted presence/absence of a stimulus of personal value. Hypotheses can be generated about these events that can be tested, like hypotheses concerning motivation, empirically and/or via owner history. The systematic inductive process for the formulation of hypothesis and deductive testing based on falsification are in accordance with the scientific method. Thus, although unknowable internal psychological states are inferable; the approach is scientific in its epistemology, emphasizing the probabilistic nature of knowledge. Thus, diagnoses are always tentative and never definitive, since they are based on inferred internal states, and they are continuously being tested and reviewed as new information comes to light, using hypothetico-deductive principles common to the scientific method. However, in contrast to the other two approaches it rejects the comprehensiveness of physical reductionism as a framework for describing and understanding animal behaviour [33]. While all biological phenomena have a physical cause, (and veterinary education is perhaps increasingly focused on this, by emphasizing the importance of identifying biological markers of disease) there is a risk of oversimplification if one focuses just on this when it comes to the complexity of the brain and factors regulating animal behaviour and thus its management. Cobb [34] presents an excellent illustration of this in relation to our understanding of the brain and brain states.

It is important to be aware of these philosophically different perspectives as they result in practical biases. For example, a medical approach to problem behaviour will tend to emphasize the importance of, and need for, chemical intervention (usually drugs) to augment appropriate psychological states. This is because it views problem behaviour largely in terms of the output of discrete disorders or pathologies which require correction (e.g., Overall [35]). The identification and definition of specific disorders is a requirement for the licensing of a drug by both the European Medicines Agency and Food and Drug Administration in the USA and this perhaps explains, at least in part, its popularity. This raises further issues over the limitations arising from this perspective and the emphasis given to Evidence Based Medicine (EBM) which are discussed further below. By contrast a behavioral approach by viewing an animal’s behaviour largely in terms of it being a response to environmental contingencies [36], emphasizes the need for these environmental cues to be rearranged for effective treatment; the epitome of this is illustrated by the use of Applied Behavior Analysis for the management of problem behaviour, e.g., by Pfaller-Sadovsky et al. [37]; Waite and Kodak [38].

Experience from the human field has shown that both the medical and behavioral approaches can be morally problematic, and there is perhaps insufficient debate of these moral concerns within the equivalent veterinary behaviour field. For example, the medicalization of human thoughts into diseased states has been used to justify the removal of patient autonomy or opinion in the selection of treatment, resulting in enforced institutionalization and sedation or the use of dubious forms of aversion therapy including electroshock [39]. This problem is conflated by the power imbalance between the clinician and client that is often associated with this model [40], which has led to a recognized need to move towards better, shared decision making to allow personalized care [41,42,43]. These concerns are equally justified when the patient is an animal and the owner is the client but also the agent for the animal’s care; for example, owners may feel pressured by the perceived authority of their veterinarian to accept the recommendation of chemical intervention on their animal without adequate consideration or discussion of alternatives. Likewise, the focus on achieving behavioural conformity through the techniques of applied behavior analysis, without full consideration of the internal state of the patient, has led to some concerns over the psychological impact of some of the routines that may be advocated even when they are associated with positive reinforcement [44]. Within clinical animal behaviour, a behavioral perspective could mean not only that the importance of depressive states might be overlooked since they are unlikely to be associated with behaviour that is problematic to and thus a cause of concern for many owners [45], but also that there might be a tendency to over-use external reinforcement which might result in reflexive stimulus-response habits based on verbal cues, devoid of the pleasure associated with an equivalent behaviour that is more response outcome focused [46]. In other words the animal might respond to a cue because it has been regimentally trained to respond, but may no longer enjoy the task, even if a reward like food was used initially. These matters deserve further ethical consideration and debate. By contrast the causal focus of the psychobiological approach is on inferred internal subjective states (based on affective and motivational considerations), which are intrinsically associated with the welfare of the individual. As such, consideration of the welfare implications is implicit within the evaluation of the appropriateness of an intervention. In this context “appropriateness” is defined in terms of the evidence that the intervention is likely to achieve the desired goal, the likelihood that it will be administered appropriately and the impact on the patient’s well-being compared to the alternatives.

## 4. Uncertainty within the Scientific Method: Justified Beliefs, Personal Opinions and *p*-Values

The scientific method is justifiably respected for the quality of the process, but it is important to appreciate that there are potentially both intrinsic biases (in terms of what is chosen to be studied and thus is scientifically explored) and methodological biases (in terms of the judgements made within the process), which can go unrecognized. Greater awareness of these biases allows a more honest discussion of the meaning of research results and increases scientific progress. This mandates an appreciation of the probabilistic nature of scientific knowledge.

The uncertainty that is inherent within all scientific knowledge described above, is often overlooked; this can result in a failure to appreciate other approaches to gaining knowledge, especially with regard to non-physical phenomena. Within science, epistemology concerns the difference between justified belief and opinion. The facts of science change as our knowledge of a subject grows, accordingly scientists change their opinion in light of new evidence that contradicts previous knowledge. Science is concerned with truth, but it is not the absolute truth of facts that is its focus, it is the truthfulness of the process that has been used to evidence the beliefs that is important. Conclusions should be based on a scientific evaluation of the evidence of all reasonable explanations: a distinction that is not always appreciated in public debates concerning the quality of evidence for a particular position. Given the inherent uncertainty of science, it is not surprising that many scientists are cautious about their conclusions, even when they feel they are scientifically justified. However, the cautiousness of the scientists may be no match for the passion or zeal of those with firmly held personal beliefs, when it comes to convincing a wider audience who may be more interested in the nature of the debate than the truth of the arguments (See Garvey [47] for excellent, entertaining discussions of this topic).

Positivism is the philosophical argument that only what can be deduced using the scientific method or logical, including mathematical, reasoning should be considered true. Unless a belief can be justified on this basis, it is simply an opinion. Debate involving non-scientific beliefs are matters of personal opinion, and not explorations of truth. The transient nature of scientific knowledge does not mean that all beliefs are equal. A scientific belief is built on evidence following a defined process: the scientific method [48]. Fundamental to this method is that scientific facts must be falsifiable. If something cannot be shown to be false, then it is not a matter of science, but purely one of personal belief. We cannot show that something is true with absolute certainty; we can only show with a certain level of confidence that all reasonable scientific alternatives are not the case, i.e., reject competing explanations. The level of confidence (or uncertainty) we have in the evidence generated from a research study is often quantified in terms of the probability value (*p*-value) of the statistical test used in the analysis of related data. This is traditionally set at an arbitrary value of *p* < 0.05 or less than a 1 in 20 chance either that the results obtained came from a situation where there was no real difference or of obtaining a more extreme value (see Hubbard [49] for a more nuanced discussion of this issue). The misrepresentation and/or misunderstanding of *p*-values is an issue of ongoing concern [50] and its value has been more fully debated elsewhere [51]. It seems that sometimes both scientists and publishing editors appear to consider *p*-values and the 0.05 threshold to be an essential and immutable value for research to be of value. This is a very naïve and unhelpful opinion when it comes to scientific knowledge.

It is well recognised by researchers that many design factors (not least sample size) affect the meaningfulness of a *p*-value from an epistemological perspective. However, the importance of reflecting on this and the associated risk of type 1 (false positive) and type 2 (false negative) statistical errors, within results is perhaps less often expressed [52]. I speculate, on the basis of many reviewer/editor comments on my work, that this is perhaps because of a mistaken opinion that discussion of uncertainty is seen to somehow undermine the quality of the research. From an epistemological perspective this is definitely not the case, but from a practical perspective it might affect the chances of publication in certain journals or securing a grant, given previously mentioned biases.

It is useful to distinguish between two different types of study which both involve statistical testing of hypotheses, but which reduce scientific uncertainty to different degrees because of their methodological rigor [52]. In a relatively young clinical discipline it is understandable that many studies may be relatively limited. They are nonetheless valuable scientifically, in that they provide useful data to inform scientific debate and clinical judgement. However, these studies should not be considered definitive. They may not only be important preludes to more definitive studies, but also critical to the generation of new ideas and hypotheses [53]. Such studies might include preliminary trials and many epidemiological studies focused on the identification of potential risk factors for a given problem or treatment outcome. By contrast, more definitive research is designed to specifically test competing hypotheses. Such studies should not only adhere to the highest standards of design, e.g., double-blinded randomized controlled trials with a specific hypothesis in mind, but also be based on prior predictions of meaningful effect type and size. Ideally the protocol should also be pre-registered, e.g., Murray et al. [54]. Unless a product makes a specific medicinal claim, this level of evidence is not required by law. So it is perhaps not surprising that many companies selling behaviour-related products that might assist in the management of a problem behaviour make a business decision not to make such an investment. Although they may seek the added value that comes from a lower level scientific publication supporting the efficacy of their product. Many studies are analyzed in a way that also makes them less definitive; for example, it could be argued that results from the use of powerful multivariate analytical techniques which ostensibly seek to control for multiple co-variates should be considered more exploratory when there is no clear evidence of pre-specification, given the risk of HARKing (Hypothesizing After Results are Known), and the damage this can potentially do to scientific progress [55,56]. Hollenbeck and Wright [57] provide an excellent discussion of this issue, including when post hoc exploratory analyses may be valuable.

Just as there should be concern over the unthinking use of *p*-values, so should there be greater concern over the use or not of statistical corrections of this value, when multiple tests are undertaken. The argument in favor of correction is that the more tests we do, the greater is the global risk of a Type I error, since there are several hypotheses being tested at the same time. If enough people, roll 8 dice, someone will eventually get the improbable result of 8 sixes on their first throw, but this is not good evidence that the dice are loaded. Statistical correction should be carefully evaluated as the trade-off between reducing the chances of a Type I error and increasing the chances of a Type II error—the equivalent of failing to reject loaded dice. The relative importance of one type of error over another is a matter of some judgment, and again depends on careful scientific reflection on a case-by-case basis. A key point to consider is how definitive the research aims or claims to be [52], and we should be humble enough to accept that most research is perhaps less definitive than it might initially seem or authors perhaps claim, though still useful given the level of uncertainty that currently exists in the field. This is one of the reasons why the iterative aspect of the scientific method is so important. At a pragmatic level, the decision as to whether or not a statistical correction should be made depends on the implications of being wrong either way, which also needs to consider how definitive the study claims to be; with the practical implication of the results discussed accordingly.

The following example illustrates some of the points made so far, and the value of explaining the decisions made, in order to reduce the risk of misrepresentation or misunderstanding of the results. Mills et al. [58] describe a fully blinded assessment of Dog Appeasing Pheromone (DAP—a form of pheromonatherapy [59]) versus placebo on anxiety related behaviour in the veterinary clinic. A relatively small population of dogs was used (*n* = 15). This study used a within subjects counterbalanced design to reduce the effects of random differences in the measures taken in the two conditions which increases the risk of a Type II error in small populations (false negative). A further concern relating to this risk was a recognition that animals can express the same emotion in different ways. Accordingly, they argued that measures of inferred emotional state (if they could be shown to be reliable) may be more sensitive than behaviour for detecting an effect of this product. These states were chosen based on the proposed ability of the pheromonal mixture to increase the perceived safety of a physical environment. No correction was made for multiple testing (three measures of emotion and three of behaviour were considered) given the small sample size and thus increased risk of Type II error if such a correction was made. In summary, the measures of inferred emotion were found to be more reliable to record than the measures of behaviour, and amongst other measures the dogs were consistently found to be more relaxed when exposed to DAP. There were no significant differences in the specific behaviour measures. These results are consistent with the predictions made a priori, and precautions taken in the design and analysis as a result. Indeed, if statistical corrections had been applied some of the results concerning emotional state would still have been significant. Does this study support the use of DAP in the clinic? Yes. Does it provide definitive evidence that DAP increases relaxation in this setting? No—further studies are required, and while we showed that the measures of relaxation were reliable, we have no further evidence that this is how they felt (evidence of convergent validity), beyond anecdotal reports of those present at the time. From a clinical perspective, should we now recommend DAP to improve the welfare of dogs visiting the vet’s clinic? Yes, especially given the relatively low financial investment required and consistent evidence that visiting the vets is stressful for many dogs and the suffering that would be caused if we rejected the evidence from this study. The clinical decision depends on a careful judgement of both the quality of the evidence and a cost–benefit analysis of potentially rejecting this, including its impact on animal welfare.

At this point it is worth highlighting the ease with which a study design can return a null (no statistically significant difference) result and the associated problems when this finding is not critically appraised. Caution is always required in the evaluation of such studies, if a type II error is to be avoided. Just as it is important to look for potential confounds with an intervention when a positive result is found, so it is important to look at experimental design when a negative result is found. As mentioned above, scientific progress is incremental and it is important to build on the insights gained from previous studies when designing a new one, to reduce the risk of inaccurate conclusions. For example, the study described previously involving DAP in the clinic [58], also noted a marked carry over effect among the dogs who were tested with DAP first. Unfortunately, this was not recognized in the design of a recent study hoping to examine the effect of DAP on the response of dogs to separation [60]. In this study, dogs were exposed to DAP and the authors report they could find no convincing evidence of a statistically significant effect of DAP. The order effect might be important to this result, as well as other methodological issues, such as: the very small sample size (just 10 dogs); the decision not to use a composite measure of inferred state despite this potentially being a more sensitive measure; the application of statistical corrections for multiple testing and the use of focal instantaneous samples every 30 s despite continuous recording of the procedure being undertaken, which would have allowed the occurrence and duration of all instances of behaviour to be calculated. All of these factors increase the risk of a Type II (false negative) statistical error.

No study is perfect, and any study can be criticized, however scientific critique considers the likelihood that any flaws have a meaningful impact on the conclusions drawn and how definitive they might be. In the latter case, they clearly do; the conclusion drawn by the authors that “*the application of a DAP diffuser did not markedly influence the behavior*” is not supported by the study, given the methodological flaws—it was simply not designed adequately to draw a specific conclusion about lack of effect. The failure to find significant effects does not provide good scientific evidence one way or the other as to whether DAP is effective in this context, since it could plausibly be argued that multiple aspects of the design increased the risk of a Type II statistical error. Given the design limitations, only the finding that there was an effect would have added much scientifically, i.e., despite these risks there was still an effect found. In other words, multiple negative results from poorly designed studies do not provide good evidence that an intervention is not effective; they simply do not increase scientific knowledge. It is for this reason that such poorly designed studies should be rejected from any quantitative synthesis of results from multiple studies, such as a meta-analysis. There is growing interest in the value of such syntheses, as they are considered to potentially offer a high level of evidence about the true effects of an intervention, but as we will see in the next section, this is not necessarily the case and there are many issues with evidence-based medicine which do not appear to be widely appreciated yet by this field.

There is also a tenuous link between statistical significance and clinical significance, which is not always understood in the applied sciences. From a practical clinical perspective, as discussed below it may be more valuable to have an estimate of the effect size of an intervention (and our confidence in this) than its *p*-value [61]. In other words how big is the effect and what is the margin of error around this? Non-overlapping confidence intervals can still be used to imply a significant effect, even without a formal test [62].

## 5. Evidence Based Medicine versus Scientific Practice

While the increased use of scientific evidence to inform clinical practice has undoubtedly brought enormous benefits, this is not the same as practicing Evidence Based Medicine (EBM). Somewhat like the importance placed on *p*-values by some scientists discussed in the previous section, I will argue in this section that some practitioners and researchers of EBM are making unsound epistemological claims about scientific knowledge.

Within the medical field there are various forms of hierarchy used within “evidence based medicine” (e.g., The Oxford Centre for Evidence-Based Medicine hierarchy [63]; Table 1) to define higher quality evidence. However, the simple claim that the strength and objectivity of the evidence increases as you ascend the hierarchy is at best misleading, and potentially false [64]. Indeed, as Borgerson [64] highlights, the concept of such a general ranking of evidence is very alien to a traditional scientific paradigm. It is therefore worth appreciating where this idea came from, and its philosophical underpinnings, to ensure it is applied appropriately. Accordingly, in this section some of the limits and concerns of EBM, that have been expressed in the human literature will be reviewed within the context of clinical animal behaviour practice, before a discussion of specific examples of poor practice within the guise of better science through an evidence based medicine approach.

What is widely considered to be the commonly applied EBM approach referred to today, is generally considered to have its origins in the text “Clinical Epidemiology” of the 1980’s, produced by academics at McMaster University in the USA [65]. This saw epidemiologists apply their skillset and expertise away from public health and towards patient care in a clinical setting. However, it is important to appreciate that clinical practice has always been evidence based; the development of EBM as a discipline really reflects a shift in the conceptualization of what constitutes evidence—away from the professional opinion of clinicians in practice and towards a more general consideration of the scientific literature. However, the evidence needed to support public health policy necessarily is focused on information about population averages, overall cost–benefit analysis etc (nomothetic knowledge), while patient care depends on recognition of individual features (idiographic knowledge), and so the application of the former methods to the latter situation requires careful scientific reflection. Failure to appreciate this difference in the knowledge required for good population versus individual patient level medicine is becoming increasingly apparent with the growing demand for more patient-centred medicine, which is focused on personal care [66]. In other words, the principles of EBM should not be applied to patient care, nor appealed to as better science without due consideration of its underlying philosophy, and the implications of this in a given context. I therefore begin with a review of six general concerns about the limits of EBM which have been discussed previously by Lambert [67] and how they might apply to advancing the scientific basis to clinical animal behaviour.

1. *The incommensurate nature of population evidence and individual patient profiles*: in other words the results of clinical trials cannot simply be applied to individual patients. In clinical animal behaviour practice, the pet owners recruited onto a trial are often highly motivated and fully engaged with a standardized treatment [68]. These individuals may not be an accurate reflection of the wider population [69]. Indeed, much of the evidence being produced to support EBM comes from academic settings which may not be representative of first opinion work where most management is undertaken [70]. There may also be geographic differences possibly due to different cultural practices that need to be considered, for example feline behaviour referral caseloads differ by country [71]. In other words, what applies on average to a population recruited to a specific clinical trial may be of limited relevance to the care of a specific patient, who has a unique profile and clinical history.

It should also be noted that most statistical methods were developed with the initial assumption that the sample was randomly drawn from the target population, which is probably unrealistic in the human or veterinary health setting. Even in a well-designed, controlled study with fully randomized allocation of treatments, the researcher does not have access to the profile of all individuals making up the target population (i.e., with a specific problem) and so cannot randomly draw on participants. This means that the error of any method could be even greater than is supposed by the analytical method used. In a non-random sample, e.g., with volunteers, there is no guarantee that the sample profile actually represents the target population profile. This point is important to appreciate, as any reasoning that aims to control for a type error I at a level of 5%, including any kind of corrections, is also affected by potential error in the sampling method.

These limitations to the generalization of findings need to be acknowledged and critically evaluated to progress beyond a position of personal opinion to the development of scientifically justifiable beliefs about best practice based on the wider scientific research available.

2. *Bias towards single/general interventions*: EBM is biased towards what can be readily measured, and so favors relatively simple interventions that can be incorporated into the “gold standard” double-blinded randomized controlled trial. This aligns EBM closely to a medical model and interventions that conform to this philosophy, but also the problems inherent within this approach when it comes to clinical animal behaviour, which were discussed above. This means EBM is well suited for, but also biased towards, interventions such as drug therapies with simple measurable outcomes. Complex, multimodal interventions or heterogenous personalized care programmes are perhaps the norm in clinical behaviour management, as both the patient and its carer typically need to engage in some form of behaviour change [72,73]. However, such interventions are not well served by the research mechanisms described in the EBM hierarchy of evidence (Table 1). Even if we focus just on the change in the animal we need to recognize that the goal of treatment is not to eliminate the underlying psychological state (like you would a pathogen) or a specific behaviour (behaviours are an intrinsic part of the repertoire of an individual), but rather to moderate it; for example by altering the probability of it being expressed at a certain intensity in given circumstances. Problem behaviour reflects clusters of degrees of behaviours, emotional states, and cognitions which arise from the complex integration of vast amounts of information [74] (i.e., they are not simply stimulus response associations [75]) and so it is not surprising that there are many interventions which may help to improve the situation, by nudging them towards a positive outcome [76]. Indeed the importance of the systematic use of “nudging” is widely recognized in human behaviour change strategies [77], but seems to be completely absent from the research literature relating to clinical animal behaviour. In short, nudging involves acknowledging that nearly everything matters, and we are subject to a lot of subconscious influences in our routine decision making. Accordingly, we can manipulate the environment to encourage certain choices without an obvious incentive or cost. Thus, the way we frame the choices that need to be made by a client can encourage a certain outcome without persuasion. For example, the inclusion of a very difficult behaviour management option can nudge a client towards accepting an alternative, but still demanding task. Likewise demonstrating a particular technique can nudge a client towards adopting it over another that is purely discussed in the clinic. The value of nudging deserves further consideration, despite the challenges associated with its evaluation, see Hummel and Maedche [78] for a useful review of evidence and issues associated with assessing the effectiveness of nudging.
3. *Exclusion of clinical skills from medical practice*: EBM through its requirements for standardization finds it difficult to accommodate professional clinical judgment, or “the art” of clinical practice. As mentioned above, each patient is unique and subjects are generally believed to perform better with a bespoke behaviour modification programme, e.g., Blackwell et al. [79]. It is difficult to comprehensively capture all the elements that need to be considered to make better decisions, for example a recent article on the role of pain in problem behaviour [80] highlights many presenting signs that need to be given consideration (note they are not proposed to be diagnostic). This work is clearly of practical use to clinicians in a field where there is so little information, but it would be considered of very low quality from an EBM perspective as it largely a series of observations. These qualitative studies or case reports (which should not be trivialized by referring to them as simply anecdotes), nonetheless, reflect the experience of many of the most qualified clinicians in the field and help to address an important gap in current professional practice.
4. *Production of formulaic guidelines*: a corollary of the above is the tendency of EBM to result in the production of clinical guidelines, which while useful can become proscriptive, limiting clinical autonomy and client choice as a result. As mentioned above, within clinical animal behaviour, protocols are usually bespoke, and this has been found to be of greater benefit to patients [79].
5. *Failure to consider client views*: What can be shown to work in clinical trials may not be what clients prefer. The four core ethical principles of clinical practice refer to autonomy, beneficence, non-maleficence and justice [81]. EBM prioritizes clinical effectiveness in the published literature, and this can potentially be at the expense of client choice or autonomy. Many behavioural interventions require the instigation of complex programmes of action and behaviour change by clients and engagement with this can be a challenge. This can be improved by increasing client autonomy in the situation for example by engaging them in intervention recommendations and decisions [82].
6. *Difficulties in translating evidence into practice*: Even when EBM produces strong evidence of best practice, then there can be issues with EBM being adopted by practitioners. Indeed, within the veterinary field there does not seem to be a relationship between the quality of research as determined by EBM and its uptake [83]. Although the practice of clinical animal behaviour has undoubtedly become more professional, this does not mean it has necessarily become more scientific. As highlighted already and below, unless practitioners and researchers become more expert in their reading and evaluation of the scientific literature, there is a danger of poor recommendations masquerading as best practice. Interestingly, EBM was developed as a pedagogical model for practice, i.e., how to draw on best evidence, but at some point it appears to have become transformed into a model of practice.

Somewhat ironically, but also possibly most damningly, Lambert [67] highlights the lack of evidence that the practice of EBM actually improves outcomes for patients.

While the hierarchy purports to illustrate increasing quality of evidence from the associate types of study, this is not necessarily the case, especially when the study does not adopt appropriate standards. Systematic reviews are generally placed at the top of this hierarchy, but that does not mean their conclusions are necessarily accurate since they might be biased. The reporting of the systematic element of the review process should allow detection of errors including systematic biases and consideration by future readers, even if it is not recognized by the original authors. Unfortunately, subsequent critical evaluation of such publications for important errors does not seem to be happening in the veterinary behaviour literature, with the consequence that poor conclusions are potentially being perpetuated. For example, Frank et al. [84] in their systematic review of the evidence for pheromonatherapy, state “*studies in which the owner of the company that produces the commercially available canine and feline pheromones was a coauthor were excluded*”. There is no scientific justification for this; although it might be argued that this exclusion avoids any risk of bias from someone with a vested interest in the results, this should be detectable from the scientific report, unless the authors believe that these authors are dishonest. This would be a very serious accusation that would have to be taken seriously (and I should point out, for which there is no evidence that I know of). If we follow the logic of these authors, then we should exclude the data from any study published by the license holder of any pharmaceutical product. This line of thinking would totally undermine the evidence-based approach, as not surprisingly those with an interest in a product are most likely to invest in the higher quality research studies which lead to the evidence required for licensing. Unfortunately, Frank et al. [84] did not employ any of the recommended methods to assess bias, e.g., funnel plot of effect sizes [85,86] and so introduced systematic bias into their own work. Such issues are compounded when the number of available studies is small, since such systematic bias (exclusion on the basis of authorship) will inevitably reduce the number of studies available for review and thus increase the risk of a false negative conclusion, i.e., that there is no strong evidence in favor of the intervention. This is another example of how researchers can either consciously or unconsciously undermine the value of a scientific approach to clinical practice.

Another way to eliminate potential bias, which was unfortunately not adopted in this particular systematic review [84] would be to undertake some form of meta-analysis on the synthesized data; without this, interpretation of the quality of the evidence remains subjective. Indeed, a subsequent meta-analysis of treatments used to control urinary spraying by cats, which did account for these factors [87], found quantifiable evidence in support of pheromonatherapy for reducing urine spraying. Thus the review of Frank et al. [84] is not of higher quality by virtue of being a systematic review, rather its results are simply more reproducible, and that includes the ability to reproduce the same bias, quite apart from other errors in the study and its failure to meet normal scientific standards of a systematic review [88,89]. These criticisms have been made in the form of letters to the journal, and whilst they can be found in academic databases such as Google Scholar, they are easy to miss. Indeed they have only been mentioned and thus considered by three of the more than 100 papers [90,91,92] that have since cited this original study. Unfortunately it seems that the original conclusions of this paper have been left unquestioned and recycled without acknowledging the concerns raised subsequently, e.g., Lloyd [93]; Williams et al. [94]; Riemer et al. [95]. Perhaps most worryingly, from a scientific perspective, this uncritical recycling includes articles which purport to be trying to assist with ethical decision making in practice, e.g., Yeates et al. [96], or promoting high standards through using an evidence based approach, e.g., Hewson [97]. The consistent failure to critically review and reflect as required by the scientific method seems to highlight either poor awareness of relevant literature by authors or selective use of references to support personal opinion (confirmation bias); neither of which help to develop best practice. Indeed, somewhat ironically, it could be argued that what is being held up as an evidence-based approach or high quality evidence is nothing more than personal opinion, which is considered one of the lowest forms of evidence. There is a need for more open and honest discussion of the limits of research studies if we are not to inadvertently foster poor scientific practice with real world impact. For example, Beck [98] has highlighted how the European Advisory Board on Cat Diseases [99] in their consideration of stress reduction recommendations seemed to simply repeat the dubious conclusion of Frank et al. [84] concerning the lack of evidence of efficacy for feline facial pheromones to reduce stress in cats without acknowledging the problems with this study and wider evidence available; nonetheless, they were willing to promote other forms of enrichment with no evidence to support them.

A further entertaining illustration of the practical problems with an uncritical approach to EBM has been highlighted by Smith and Pell [100]. They undertook a systematic review of the evidence from what would typically be considered higher level evidence studies (randomized controlled trials) that parachutes prevent serious trauma or death when jumping out of a plane. Needless to say, they found no evidence to support this and to make their point concluded “*As with many interventions intended to prevent ill health, the effectiveness of parachutes has not been subjected to rigorous evaluation by using randomised controlled trials. Advocates of evidence based medicine have criticised the adoption of interventions evaluated by using only observational data. We think that everyone might benefit if the most radical protagonists of evidence based medicine organised and participated in a double blind, randomised, placebo controlled, crossover trial of the parachute*”. Yeh et al. [101] took this example a step further, to highlight further flaws in the approach, by conducting the first randomized control trial to address the issue identified by Smith and Pell [100]. From 92 people approached and asked if they would be willing to jump out of the plane at its current altitude 23 agreed. For ethical reasons the trial was not blinded. There was no difference in the risk of serious of injury between the two groups. They acknowledge limitations, such as the lack of blinding, and also identified a difference between individuals screened but not enrolled: those that chose to take part were in an aircraft at significantly lower altitude (mean altitude 0.6 m) compared to those that did not (mean altitude: 9146 m) and travelling at a lower velocity (0 km/h vs. 800 km/h). In other words, the only people who chose to take part were in stationary planes on the ground! Whilst this might seem to be a facile example, the important point here is to appreciate how such results would be interpreted using the common principles applied in EBM. They rightly caution that these results might not generalize to planes moving at higher altitude, but the only evidence to date at the level of a randomized controlled study, indicates that parachutes provide no benefit when jumping from an aircraft. Thus, if we were interested in value for money, and simply prioritized treatment on the basis of evidence and cost, you would be forced to conclude that you should not invest in parachutes! Although this is not a conclusion that would be drawn by any rational thinking individual, it highlights the dangers of the uncritical application of the hierarchy of evidence or unthinking recycling of its conclusions. It may simply be used to justify one’s own biases, and lose it objectivity. Indeed Goldenberg [102] argues that “*The appeal to the authority of evidence that characterizes evidence-based practices does not increase objectivity but rather obscures the subjective elements that inescapably enter all forms of human inquiry*”. As noted already, from the veterinary behavioural medicine perspective, available evidence might suggest that greater treatment success is often associated with the inclusion of ideographic knowledge of the patient that allows the development of an individualized programme [79]. Accordingly, there is a need to be cautious about placing too much emphasis on the nomothetic knowledge derived from population level studies on which much EBM is traditionally based. It is important to appreciate the limits of the applicability of these findings to exactly the type of individualized treatment programmes we may be striving to create [11,103]. This does not mean we should abandon a scientific approach, rather we must preserve it by ensuring we avoid any temptation to reduce it to a simple, unreflective, formulaic process.

## 6. Applied Behavior Analysis and Evidence of Intervention Efficacy

By contrast, within Applied Behavior Analysis, the uniqueness of the individual is very much at the centre of treatment and so scientific methods for establishing efficacy have focused on addressing the challenge of statistical confidence when n = 1. In their classic paper “*Some current dimensions of applied behavior analysis*” Baer et al. [104] emphasize the importance of careful observation and measurement to establish the efficacy of an intervention. They highlight two methods that may be used to help demonstrate this:

1. reversibility, i.e., the consistent return to the base state once an intervention which brings about a meaningful change is withdrawn; and
2. the use of multiple baselines, in which baseline levels of response are established across a variety of contexts and the intervention introduced in one of these. A relevant change should change the baseline of the behaviour only within the context that it has been introduced. The process can then be extended to other contexts and similar changes should occur. This negates the need to withdraw treatment which could be practically and ethically problematic.

With sufficient repetition, these results can be made more robust through appropriate statistical analysis. This approach has been adopted in a number of recent reports concerning the application of functional analysis within the field of clinical animal behaviour (e.g., Dorey et al. [105]; Winslow et al. [106]; Pfaller-Sadovsky et al. [37]). While these methods may be used to establish efficacy of a given intervention in a specific context, their specificity means that their generalizability to other contexts may be limited and certainly should not be assumed. This is not least because a behaviour may have a completely different functional basis depending on its context. For example, an animal that runs from A to B may be running towards B (due to some incentive at B) or away from A (due to some aversive). Accordingly factors which might alter running behaviour in one context might have no effect or even the opposite effect in the other. Similar limitations apply when trying to predict the value of some form of adjunct (i.e., supplementary aid, such as a drug, nutraceutical, pheromone product, etc.) to the behaviour modification exercises. Like a reinforcer, the value of an intervention is defined post hoc, i.e., by its effect and so a behavioral approach provides a limited rational basis for the selection of one adjunct, or indeed any intervention, over another a priori in a novel context, especially when knowledge of the role of underlying psychological factors is uncertain. Thus while there is much scientific merit to the behavioral approach, it has serious limitations when it comes to considering the development and selection of specific interventions in advance.

## 7. Insights from a Psychobiological Perspective

As mentioned earlier, the psychobiological approach seeks to embrace the best elements of scientific practice associated with both a medical approach, including evidence based medicine, and applied behavior analysis. This includes best practice in the execution of research; however the psychobiological approach places a different emphasis on what is important to measure. If, as stated earlier, a problem behaviour is first and foremost a human perception and/or an inferred internal state controlling behaviour that needs to be treated, then the primary outcome of interest, when considering the efficacy of an intervention, is owner satisfaction and/or perception of the animal’s state. This type of outcome incorporates not only the potential efficacy of the intervention of interest but also its implementation in practice. Historically, by contrast, in pivotal clinical trials aimed at product registration [107,108,109,110], owner satisfaction has tended to be a secondary outcome, with the primary focus being on what is considered more objective measures such as behaviour. This is perhaps because of the physical reductionist bias of regulatory authorities, discussed earlier. While behavioural measures may be useful, even invaluable, a psychobiological perspective highlights additional important measures which can enhance appreciation of the practical value of the work. A comparison between clinical behavioural results and client perceptions from one of the aforementioned studies illustrates this point further. King et al. [107] set the standard for studies of treatment success for separation anxiety in dogs, by defining treatment success as a given sign having disappeared or improved; i.e., the latter refers to the proportion of the population that improved to some degree, not the amount of improvement. From a practical perspective, a dog might urinate less or even be less destructive, but if the behaviour is still occurring with sufficient frequency to disturb the owner, this is unlikely to be a satisfactory outcome for a client. Accordingly, we should focus on the measures which are likely to have most meaning to clients. If we consider the proportion of animals for whom destructiveness disappeared at the recommended dose of 1–2 mg/kg clomipramine every 12 h the figures are 19% at 28 days, 40% at 56 days and 53% at 84 days (the figures for the other behavioural signs are all lower on day 84); by contrast the owner’s global assessment of their dog as having been cured of separation anxiety is 0%, 12% and 27% for these time periods. Other categories in the owner’s global assessment of improvement are little, moderate and much, but unfortunately we do not know how this relates to owner satisfaction.

By contrast, more recent pivotal studies aimed at product registration for noise fears in dogs [109,110] appear to have taken a more psychobiological approach, with the primary outcome described as “*owner assessment/rating of overall treatment effect*”, with reference to anxious/fearful behaviour, with behavioural measures used as second co-primary variables of interest. Both of these studies also express results in terms of odds ratios, which are more clinically informative. Moreover, they also use some real world measures of treatment outcome relevant to owners (e.g., timepoint when the owner felt the dog could be left alone [110], the usability of the product [109] and success of treatment).

Thus, while the four studies discussed in this section are of equal standing from an evidence-based medicine perspective (as RCTs), it could be argued that the quality of the latter two studies is better, due to the content of the results presented. Further consideration of the issues raised here, will hopefully continue to improve the reporting of scientific results in veterinary behavioural medicine. Unfortunately, as we have already seen, few of the potential interventions for problem behaviour management have such a strong scientific basis. Therefore, in the final section of this manuscript a framework is provided to assist with clinical decision making, drawing on the points discussed so far relating to the effective use of available scientific information.

## 8. How Do We Make Best Use of Available Scientific Information to Improve Clinical Decision Making?

In order to make best use of available scientific information it is important to appreciate and accept the uncertainty inherent within all such information. Nothing is absolute, but that does not mean all information is equal or scientific information is no better than personal opinion. Scientific literacy means being able to recognize what can be taken from a publication to help reduce our ignorance and what cannot be logically deduced. Critique requires a judgment on the likely impact of flaws within a design (since no study is perfect) on the conclusions that can be drawn, and should be distinguished from simple criticism, which seeks to point out the flaws. Evaluating the quality of a study does not just come from the design but also how it is executed and so it is important to evaluate the methods carefully and ensure valuable information is available. To this end, the growth of open access publication and open research agreements aimed at encouraging data sharing (e.g., the policy of public funders like UK Research and Innovation to include the Concordat on Open Research Data [111]) are potentially important advances to supporting better use of available data. Researchers should also be encouraged to use open access repositories to deposit anonymized data (e.g., Center for Open Science [112]), to allow further review, and/or analysis. The inclusion of raw data appendices to online publications allowing further analysis is also helpful in this regard. A report is simply the authors account and evaluation of the data; this does not mean this is the limit of the information, nor necessarily that the emphasis they give and conclusions they draw are necessarily correct. Another consequence of the growth of open access publications is not only increased public accessibility, but also a need for greater scientific literacy to ensure that scientific information is not misrepresented, but used to maximum benefit. This requires that clinicians have a high degree of scientific literacy skills so that they can effectively utilize scientific information to reduce uncertainty and make better clinical decisions to the benefit of their patients. Accordingly, I provide below some points for readers to consider, which aim to complement the information provided above and provide a point of reference for evaluating the content of scientific publications, highlighting common ontological and epistemological errors which may result in poor clinical use of the information. The importance of these points should be apparent from the earlier sections of this article. I do not address the issue of the scientific quality and limitations of specific designs [113] and statistical analyses used in studies [114], which is generally covered in traditional scientific training, and widely covered elsewhere.

### 8.1. Be Mindful of Assumptions about Terminology and the Implications of This

Those reading publications should not assume that their definition of a problem behaviour/client complaint (e.g., separation anxiety) is necessarily the same as that of the author. We should look carefully for the definition used by authors (who likewise should ensure they carefully define such terminology). Depending on the nature of the research definition might be done in several ways, for example through the description of inclusion and exclusion criteria or by reference to a diagnosis which ideally makes reference to the context of the behaviour, and associated motivational and emotional states. The process used to make inferences about these latter two states should also be outlined, as appropriate. Critically it is important that any consideration or discussion of results in relation to the wider scientific literature, remains mindful of the potential that the populations being studied may be quite different, even if they have the same label. This will prevent the misrepresentation and overgeneralization of the findings.

Likewise, it is important to recognize how treatments are defined and the limitations of this. Some studies, e.g., Riemer [115] in a survey of treatment of fear of fireworks, depend on public understanding/definition of these treatments. This assumption about public knowledge can result in unsound conclusions, for example many behaviour clinicians will be familiar with owners misdescribing supplements as medications or various plug-in devices as pheromones. Indeed since the commercial use of the term pheromone appears to be unprotected, many products with no pheromonal basis from a scientific perspective may make reference to pheromones. This lack or protection may lead to many unrelated products being described as pheromone-related. For example, “*Broadreach Nature Relaxing Moments calming room spray for dogs*”^TM^ describes itself as “*a special blend of fragrance extracts to simulate canine pheromones*” but closer inspection of composition reveals only Valerian Root Extract, Chamomile Extract, Rosemary Oil, Clove Oil which are herbals and not pheromonal with no logical reason as to why these should be considered to simulate pheromones [116]. Unfortunately, this issue of clients not knowing what is and is not a pheromone product is not recognized by Riemer in her paper, and so her conclusion that “*… it seems likely that a placebo effect accounts for the perceived effectiveness of those products where the success rates were 35% or less in the present study, which was the case for pheromone products*…” is unsound, especially in light of more direct evidence to the contrary, e.g., Landsberg et al. [117]. To refer back to the principle outlined above in the section “*Uncertainty within the scientific method: the place of justified beliefs versus opinions and the meaningfulness of p-values*” it is worth emphasizing from a clinical perspective only a positive result would be of interest. A negative result does not help to reduce uncertainty given the flaw described.

### 8.2. Recognize the Limits of Methodological Design Given the Proposed Mechanism of Action

It is obviously important that any proposed intervention has a rational scientific basis, but by the same measure the method used to evaluate it needs to be rational. As discussed above, simple dependence on the hierarchy within EBM as a measure of quality can lead to unhelpful conclusions. The rationale for a particular methodology will be affected by pragmatic factors like, how much we already know about an intervention based on both research and both clinical experience. Randomized controlled trials are an essential requirement for the registration of medications and are expensive. They also imply a medical perspective on treatment and associated limitations, as described above. Not least, in the case of clinical animal behaviour these sorts of trial often refer to the effect seen in a population that has been very loosely defined on the basis of superficial behavioural characteristics (e.g., King et al. [107]; Simpson et al. [108]) which describe a syndrome, rather than a condition with a validated methodology. This definition of a general syndrome might explain why, the aforementioned studies and others using similar criteria to define separation anxiety, consistently report no change in around 15% of subjects. This finding suggests a lack of specificity in the proposed diagnostic process (which makes no attempt to assess internal constructs like motivational and emotional state) and that 15% of subjects expressing the signs do so for reasons unrelated to the proposed treatment plan. Trials also necessarily refer to average population effects (who may not be representative of the population seen in general practice) and thus nuances associated with the individual are typically lost. These factors deserve careful consideration. This is where reports of case studies and case series can be invaluable, as they may offer important insight into important individual factors. Accordingly greatest clinical insight is gained not from the hierarchy of evidence but the synthesis of evidence at all levels, with appropriate caveats considered for each study design. Placebo controlled RCTs help to establish that something is better than a placebo, but this is often not the primary concern. From a clinical perspective, we are perhaps more interested in the size of effect, so we can make a reasonable cost–benefit analysis in a given case. Accordingly, we need to look beyond just the scientific quality of the metrics used in a study and also consider its practical utility. Two points are of particular note here: first how the measures relate to the problem, which is an owner perception; secondly how they relate to the well-being of the patient. These two points have been discussed to some extent above, but it is worth emphasizing that the latter consideration is essential if we wish to operate according to the highest ethical standards. There are important caveats too which we need to be aware of. As mentioned above, researchers may, for good reason, use composite measures which refer to internal state (e.g., patient anxiety levels), but it is important to keep in mind that this is often an unvalidated perception (i.e., a terminological short hand for the perceived internal state of an observer based on a diversity of behaviours). This does not mean it is not useful (so long as it is reliable, it can provide good evidence that an effect is occurring), but it does mean we need to be careful about any abstractions we make. For instance, if the term anxiety is used in this way in a particular study, this does not mean any effect seen can necessarily be expected in a different context where we believe the patient is anxious.

Having established that the experimental design and metrics used are of clinical relevance, it is useful to then consider the size of effect. In this regard, *p*-values can be deceptive for reasons already discussed, however this point deserves further considering at this point. A lack of significant difference does not imply equivalence. This is particularly important when considering comparative studies. As mentioned above it is easy to find no significant difference in a poorly designed study. Beata et al. [118] reported on the effects of alpha-casozepine versus selegiline on anxiety disorders in dogs, and claimed on the basis of their data that “*the effects of treatment with selegiline and alpha-casozepine … were found to be equal…*”. However, there are several useful scientific lessons to take from this study, which, to its credit, provided the necessary data to show how inappropriate was the conclusion. First as already mentioned a lack of significant difference does not imply equality and this is particularly the case given the design of the study, so a claim of equivalence such as the “same” should not be claimed unless an a priori calculation has been made. In fact, from a statistical perspective any claim of equivalence must be stated in relation to the size of a difference considered equivalent and the likelihood of this given the variability of treatment effects—this typically means several hundred subjects might be required. In this case, the sample size was very small (19 dogs in each group), and so the likelihood of failing to show a significant difference was very high. The risk of a false negative is further increased, when it is realized that nearly half the subjects (18/39) were not considered treatment successes. This not only reduces the power even further of being able to detect a difference in those cases where the intervention seemed appropriate, but also questions the validity of the diagnostic instrument used (Evaluation of Dogs Emotional Disorder- EDED scale) to define anxiety disorders, given its poor sensitivity in predicting the effect of therapeutic intervention. Using such a poor diagnostic instrument to define the target population only further increases the chance of a false negative result in a comparative study. Accordingly, I would suggest that these flaws mean a more appropriate conclusion would be along the lines of: “*based on the sample used and considering all limitation of this study we did not find significant difference between the two groups and cannot evaluate if they have any degree of equivalence*”. However, because the data are well reported we can take home some useful insights, regarding the poor quality of the EDED scale as a tool for predicting treatment success when either agent is used. The data on individual subpopulations might also be useful pilot data for future hypothesis testing, if they could be examined further to identify the potential differences between responders and non-responders in each group.

In general, metrics of effect sizes, such as Cohen’s d, Hedges g or simply odds ratios with confidence intervals e.g., Engel et al. [110] are often of more value to clinicians than *p*-values. However, this should not be taken to mean that *p*-values are not of use. The presentation of both provide the richest information for discussion of clinical significance. Even if a study does not reach the statistical threshold for significance, it may still show a large effect size, especially if the sample was small; which might provide valuable preliminary insight into the potential value of an intervention; especially in a field like clinical animal behaviour where there are so few high-powered studies. It is perhaps unrealistic to expect this situation to change much, with no regulatory requirement for the demonstration of efficacy of interventions within clinical animal behaviour.

### 8.3. A Hierarchy Scientific Relevance for Clinicians

Neither assessing evidence quality nor applying this assessment to real world clinical situations in a meaningful way lends itself well to simply formulaic solutions. As mentioned already, scientific advance is about reducing uncertainty and it is important to keep this in mind when considering the information we have available to assist clinical decision making. At both a pragmatic and scientific level, we can still make rational use of poorer quality information, so long as we engage with the essential scientific skill of critical reflection. This means we must carefully consider the importance of identifiable flaws in line with the principles described in this paper. Another important principle is to be open to revising our conclusions as new information comes to light. This information may come from new insights into existing evidence or new evidence. We might then be able to rationally justify one intervention over another, even when evidence is far from ideal. I suggest from both a practical and scientific perspective this is preferable to simply dismissing poor quality evidence so long as it is not fatally flawed.

I describe below a “logic” hierarchy to aid the selection of the best adjuncts to behaviour modification exercises given this imperfect reality. It is assumed that the behaviour modification exercise is effective but would benefit from augmentation. Level 1 represents proximity to the ideal, with higher numbers indicating less desirable options; at each stage the significance of imperfections has to be considered in relation to the patient under consideration, so that treatment can remain personalized. Through critical reflection of published data (rather than simple acceptance of author conclusions), we can make best use of the available evidence to reduce our uncertainty about the value, or potential value, of one intervention over another on a rational basis. Imperfect evidence is still preferable to no evidence, so long as the costs of it being wrong are less than the costs of ignoring it if it is right. An essential practical point concerning the use of this hierarchy is careful reflection on the impact of our decision about the evidence being wrong. This reflection is important up to and including the point, when we decide to prefer one intervention over another, in order to avoid the literally fatal flaws associated with the evidence concerning parachutes described earlier.

Level 1. An adjunct with strong evidence of meaningful efficacy in relevant diagnostic contexts (including representative populations) from diverse well-designed trials, ideally supported by additional insight relating to the specific characteristics of the patient under consideration, to allow confidence in the personalized treatment plan.

- Ideally the diagnostic context should include a rational defense concerning inferences about the motivational and emotional state underlying the behaviours of concern.
- The specific risks, e.g., health risks associated with any intervention need to be considered and can result in contra-indication.

Level 2. An adjunct with consistent evidence of meaningful efficacy from multiple appropriate studies (beware the poorly designed study which shows a negative result), which may have flaws (but there is not a consistent confound across all studies) in relevant diagnostic contexts

Level 3. An adjunct with some evidence or potential efficacy from a relevant study, including a case series or the documented clinical experience of multiple independent clinicians, giving due consideration to the relevance of the evidence to the current case. In the case of multiple potential adjuncts being considered then a rational argument should be made concerning the relative importance of one set of flaws and knowledge gaps over another, for each intervention under consideration.

Level 4. An adjunct whose efficacy is based on personal experience (own or others).

This proposed hierarchy provides a guide to the relative weight that should be given to the available evidence, which recognizes perhaps a greater diversity of sources, through its emphasis on critical reflection of both the quality of evidence and its relevance. In reality the scale exists on a continuum; many adjuncts will sit between levels and the merits of one over another depend on an assessment of the evidence in relation to the case being considered. This approach maintains an individualized treatment perspective. The key question to keep asking when reviewing evidence is: to what extent does this information help to reduce scientific uncertainty in my clinical decision making?

## 9. Conclusions

The scientific method remains the most powerful tool for determining knowledge, however it is incumbent upon anyone claiming to use this process to apply it honestly and to the best of their ability. This obligation is especially important when one seeks to take responsibility for translating research into practice. When undertaking such knowledge translation, it is imperative that individuals do not simply apply formulaic processes, but that they actively engage in scientific thinking throughout the process. This includes a balanced consideration of both the limitations of knowledge and its inherent uncertainty. The generation of knowledge is an iterative process and so individuals need to be careful about simply referring to original work, whose conclusions may need refining since their original publication. The advent of electronic publications, makes it much easier to track citations, including criticism, which can prove invaluable in this regard.

This article has provided a point of reference for future discussion of more effective methods for evaluating interventions within the field of clinical animal behaviour. To assist the debate, it has sought to highlight the differing philosophical underpinnings to the discipline, which are rarely articulated and often go unrecognized. It is hoped that increased awareness of the issues raised here will help fuel more informed debate, so that the undoubted value of a scientific approach can be more fully realized to the benefit of our patients.

## Figures and Tables

**Table 1:** Hierarchy from the Oxford Centre for Evidence Based Medicine, reproduced from: https://www.cebm.net/2009/06/oxford-centre-evidence-based-medicine-levels-evidence-march-2009/, accessed on 17 September 2022. RCT = Randomized Controlled Trial, SR = Systematic Review, CDR = Clinical Decision Rule, Absolute SpPin = a diagnostic finding whose Specificity is so high that a Positive result rules-in the diagnosis, Absolute SnNout = a diagnostic finding whose Sensitivity is so high that a Negative result rules-out the diagnosis.

Level	Therapy/Prevention, Aetiology/Harm	Prognosis	Diagnosis	Differential Diagnosis/Symptom Prevalence Study
1a	SR (with homogeneity) of RCTs	SR (with homogeneity) of inception cohort studies; CDR validated in different populations	SR (with homogeneity) of Level 1 diagnostic studies; CDR with 1b studies from different clinical centres	SR (with homogeneity) of prospective cohort studies
1b	Individual RCT (with narrow Confidence Interval)	Individual inception cohort study with >80% follow-up; CDR validated in a single population	Validating cohort study with good reference standards; or CDR tested within one clinical centre	Prospective cohort study with good follow-up
1c	All or none	All or none case-series	Absolute SpPins and SnNouts	All or none case-series
2a	SR (with homogeneity) of cohort studies	SR (with homogeneity) of either retrospective cohort studies or untreated control groups in RCTs	SR (with homogeneity) of Level >2 diagnostic studies	SR (with homogeneity) of 2b and better studies
2b	Individual cohort study (including low quality RCT; e.g., <80% follow-up)	Retrospective cohort study or follow-up of untreated control patients in an RCT; Derivation of CDR” or validated on split-sample only	Exploratory cohort study with good reference standards; CDR” after derivation, or validated only on split-sample or databases	Retrospective cohort study, or poor follow-up
2c	“Outcomes” Research; Ecological studies	“Outcomes” Research		Ecological studies
3a	SR (with homogeneity) of case–control studies		SR (with homogeneity) of 3b and better studies	SR (with homogeneity) of 3b and better studies
3b	Individual Case-Control Study		Non-consecutive study; or without consistently applied reference standards	Non-consecutive cohort study, or very limited population
4	Case-series (and poor quality cohort and case–control studies)	Case-series (and poor quality prognostic cohort studies)	Case-control study, poor or non-independent reference standard	Case-series or superseded reference standards
5	Expert opinion without explicit critical appraisal, or based on physiology, bench research or “first principles”	Expert opinion without explicit critical appraisal, or based on physiology, bench research or “first principles”	Expert opinion without explicit critical appraisal, or based on physiology, bench research or “first principles”	Expert opinion without explicit critical appraisal, or based on physiology, bench research or “first principles”

## Data Availability

All data are taken from published reports. No other data are included.
